# Aspect d´ovaire intra-utérin d´une môle partielle

**DOI:** 10.11604/pamj.2021.39.120.29292

**Published:** 2021-06-11

**Authors:** Karima Mekni, Chiraz El Fkih

**Affiliations:** 1 Service de Gynéco-obstétrique, Hôpital Mahmoud El Matri, Faculté de Médecine de Tunis, Université Tunis El Manar, Ariana, Tunisie

**Keywords:** Aspect, môle, histologie, Appearance, molar, histology

## Abstract

The study involved a 28-year-old female patient with no particular past medical history. At 7 weeks of amenorrhea, she presented at the Gynecological Emergency Department with pelvic pain. Clinical examination showed good general condition; vaginal examination objectified that the cervix was very far back (posterior), closed, without metrorrhagias or latero-uterine mass. Ultrasound showed fetal cardiac activity and all around the sac several “lacunae” (empty spaces) without leakage of fluid. Molar pregnancy was suspected based on image examination; hCG level was 37920 UI. The diagnosis of embryonated mole was evoked and complementary thoraco-abdominopelvic CT scan was performed to support the diagnosis and as staging evaluation. This showed partial hydatiform mole without signs of loco-regional or remote extension, with anterior intrauterine myoma. Ultrasound-guided aspiration was performed after availability of blood supply. During aspiration, vescicles were observed. Anatomo-pathological examination initially showed interrupted pregnancy with no chorionic villi. Given the strong suspicion of molar pregnancy, multiple sections were performed which showed rare large chorionic villi with edematous axis. These rare villi were lined with trophoblastic coverage of usual abundance and morphology, suggesting partial mole. The woman received effective contraception with weekly monitoring of BHCG levels. She was monitored until she experienced negative results from three consecutive tests (A, B, C, D).

## Image en médecine

Il s´agit d´une patiente âgée de 28 ans sans antécédents pathologiques notables. Elle a consulté aux urgences de gynécologie pour des douleurs pelviennes sur une aménorrhée de 7 semaines. L´examen a trouvé un bon état général avec au toucher vaginal un col fermé long postérieur sans métrorragies ni masse latéro-utérine. L´échographie trouvait un embryon avec activité cardiaque positive et tout autour du sac présence de plusieurs images lacunaires sans épanchement: c´était l´aspect d´ovaire intra-utérin. Le taux de gonadotrophine chorionique humaine (BHCG) était à 37920 UI. Le diagnostic d´une môle embryonnée était évoqué et on a fait un complément d´exploration par un scanner thoraco-abdomino-pelvien pour étayer le diagnostic et comme bilan d´extension. Il était en faveur d´une môle hydatiforme partielle sans signes d´extension loco-régionale ou à distance avec un myome intra utérin antérieur. La décision était de l´aspirer sous échographie après une réserve de sang. Au cours de l´aspiration, un aspect vésiculeux du produit a été constaté. L´examen anatomopathologique a conclu initialement à une grossesse arrêtée avec absence de villosités choriales. Devant la forte suspicion de grossesse molaire, une relecture a été formulée avec multiplication des coupes qui ont montrés des rares villosités choriales de grande taille à axe œdémateux. Ces rares villosités étaient tapissées par un revêtement trophoblastique d´abondance et de morphologie habituelles ce qui était en faveur d´une môle partielle. La femme était mise sous contraception efficace avec surveillance hebdomadaire de taux de BHCG jusqu´à trois taux successifs négatifs (A, B, C, D).

**Figure 1:**
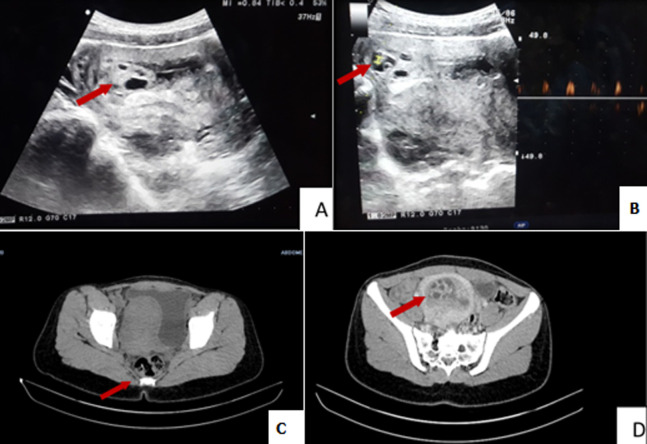
(A) aspect échographique d´un ovaire intra-utérin; (B) présence d´un embryon avec activité cardiaque; (C) aspect image radiomagnétique (IRM) en T1; (D) aspect IRM en T2

